# Ilioinguinal Nerve Neurectomy is better than Preservation in Lichtenstein Hernia Repair: A Systematic Literature Review and Meta-analysis

**DOI:** 10.1007/s00268-021-05968-x

**Published:** 2021-02-19

**Authors:** Roberto Cirocchi, Marco Sutera, Piergiorgio Fedeli, Gabriele Anania, Piero Covarelli, Fabio Suadoni, Carlo Boselli, Luigi Carlini, Stefano Trastulli, Vito D’Andrea, Paolo Bruzzone

**Affiliations:** 1 Department of Medicine and Surgery, University of Perugia, Perugia, Italy; 2 Inguinal Nerve Working Group, Terni, Italy; 3 School of Law, Legal Medicine, University of Camerino, Camerino, Italy; 4 Department of Medical Science, University of Ferrara, Ferrara, Italy; 5 Department of Digestive Surgery, AOSP of Terni, Terni, Italy; 6 Department of Surgical Science, Sapienza Università Di Roma, Rome, Italy; 7 Department of General Surgery and Surgical Specialties “Paride Stefanini”, Sapienza Università di Roma, Rome, Italia

## Abstract

**Objective:**

This study aimed to evaluate the incidence of chronic groin pain (primary outcome) and alterations of sensitivity (secondary outcome) after Lichtenstein inguinal hernia repair, comparing neurectomy with ilioinguinal nerve preservation surgery.

**Summary background data:**

The exact cause of chronic groin postoperative pain after mesh inguinal hernia repair is usually unclear. Section of the ilioinguinal nerve (neurectomy) may reduce postoperative chronic pain.

**Methods:**

We followed PRISMA guidelines to identify randomized studies reporting comparative outcomes of neurectomy versus ilioinguinal nerve preservation surgery during Lichtenstein hernia repairs. Studies were identified by searching in PubMed, Scopus, and Web of Science from April 2020. The protocol for this systematic review and meta-analysis was submitted and accepted from PROSPERO: CRD420201610.

**Results:**

In this systematic review and meta-analysis, 16 RCTs were included and 1550 patients were evaluated: 756 patients underwent neurectomy (neurectomy group) vs 794 patients underwent ilioinguinal nerve preservation surgery (nerve preservation group). All included studies analyzed Lichtenstein hernia repair. The majority of the new studies and data comes from a relatively narrow geographic region; other bias of this meta-analysis is the suitability of pooling data for many of these studies.

A statistically significant percentage of patients with prosthetic inguinal hernia repair had reduced groin pain at 6 months after surgery at 8.94% (38/425) in the neurectomy group versus 25.11% (113/450) in the nerve preservation group [relative risk (RR) 0.39, 95% confidence interval (CI) 0.28–0.54; Z = 5.60 (*P* < 0.00001)]. Neurectomy did not significantly increase the groin paresthesia 6 months after surgery at 8.5% (30/353) in the neurectomy group versus 4.5% (17/373) in the nerve preservation group [RR 1.62, 95% CI 0.94–2.80; Z = 1.74 (*P* = 0.08)]. At 12 months after surgery, there is no advantage of neurectomy over chronic groin pain; no significant differences were found in the 12-month postoperative groin pain rate at 9% (9/100) in the neurectomy group versus 17.85% (20/112) in the inguinal nerve preservation group [RR 0.50, 95% CI 0.24–1.05; Z = 1.83 (*P* = 0.07)]. One study (115 patients) reported data about paresthesia at 12 months after surgery (7.27%, 4/55 in neurectomy group vs. 5%, 3/60 in nerve preservation group) and results were not significantly different between the two groups [RR 1.45, 95% CI 0.34, 6.21;Z = 0.51 (*P* = 0.61)]. The subgroup analysis of the studies that identified the IIN showed a significant reduction of the 6th month evaluation of pain in both groups and confirmed the same trend in favor of neurectomy reported in the previous overall analysis: statistically significant reduction of pain 6 months after surgery at 3.79% (6/158) in the neurectomy group versus 14.6% (26/178) in the nerve preservation group [RR 0.28, 95% CI 0.13–0.63; Z = 3.10 (*P* = 0.002)].

**Conclusion:**

Ilioinguinal nerve identification in Lichtenstein inguinal hernia repair is the fundamental step to reduce or avoid postoperative pain. Prophylactic ilioinguinal nerve neurectomy seems to offer some advantages concerning pain in the first 6th month postoperative period, although it might be possible that the small number of cases contributed to the insignificancy regarding paresthesia and hypoesthesia.

Nowadays, prudent surgeons should discuss with patients and their families the uncertain benefits and the potential risks of neurectomy before performing the hernioplasty.

## Introduction

Inguinal hernia is one of the most common male diseases worldwide [1]. Inguinal postoperative chronic pain (PCP), also known as inguinodynia or groin pain, is identified as a pain persisting more than 3 months [2]; it is one of the most common complications after inguinal hernia repair [3–7]. The prevalence rate of PCP ranges from 0 to 63% independently of the surgical techniques used [8, 9]; this high variability is the consequence of different definitions of inguinal postoperative pain, end points of the studies, and methodologies of pain evaluation [9–12]. Inguinal postoperative chronic pain can be secondary to entrapment or stretching of nerves, inflammation, fibrotic reactions, or formation of neuromas, and it may require several interventions, including oral analgesics, local anesthesia, physiotherapy, or further surgery [13–18].

Ilioinguinal nerve section (neurectomy) has been proposed to reduce the incidence of chronic groin pain after inguinal hernia repair [10]. Thus, this systematic literature review and meta-analysis aims to evaluate the incidence of chronic groin pain (primary outcome) and sensitivity alterations (secondary outcome) after Lichtenstein inguinal hernia repair [19, 20], comparing neurectomy with ilioinguinal nerve preservation surgery.

## Methods

We carried out a systematic literature search (April 10, 2020) using PubMed, Scopus, and Web of Science. The protocol for this systematic review and meta-analysis was submitted and accepted from PROSPERO: CRD42020161015. The search terms used were inguinal nerve AND hernia, neurectomy AND hernia, ilio-inguinal nerve AND hernia, ilio-inguinal nerve AND neurectomy, and hernia repair AND nerves. Additional search was performed on gray literature on Google Scholar (https://scholar.google.com/) and Google Books (https://books.google.it/). This systematic review of literature was conducted according to the preferred reporting Items for systematic reviews and meta-analyses (PRISMA) guidelines [21] and the recommendations of the Cochrane Handbook for Systematic Reviews of Interventions [22].

All titles and abstracts were evaluated to identify those that could be included in the analysis. The inclusion criteria were as follows: studies including patients undergoing an inguinal hernia repair with mesh according to the Lichtenstein technique [19, 20], comparative studies of section and preservation of the ilioinguinal nerve, studies in which postoperative pain was analyzed at 6–12 months, and randomized controlled trials (RCTs). Pain scores were recorded using the visual analog scale (VAS): 0 being no pain and 10 being the worst imaginable pain. Pain scores were recorded either during normal daily activities or during walking if former information was available. If pain scores were recorded using the verbal rating scale (VRS), that is, no pain, mild, moderate, or severe pain, they were subsequently converted to VAS. The conversion tool was based on a prospective study and review comparing VAS and VRS in postoperative pain [23]. This neuropathic pain can be accompanied by paresthesia and hypoesthesia [24]. We have analyzed the presence of postoperative paresthesia or hypoesthesia only in studies that reported these data as separate outcomes.

The exclusion criteria include reviews, meta-analysis, clinical cases, editorial opinion articles, and non-RCTs.

### Subgroup analysis

Postoperative groin pain was assessed according to its severity using a four-point scale and assigning numerical values from 0 to 3 (i.e., 0 = nothing, 1 = mild, 2 = moderate, 3 = severe).

### Analysis method

Among the authors, R.C. and M.S., respectively, handled data extraction and bias risk analysis, subsequently comparing and expressing results based on common opinion.

### Data and statistical analysis

The meta-analysis was carried out including RCTs, which compared neurectomy and ilioinguinal nerve preservation surgery, during Lichtenstein inguinal hernia repair with mesh.

For data evaluation and analysis in the included studies, binomial aggregate prevalence estimates were defined and 95% confidence intervals (CI) were calculated using the Review Manager software (RevMan) [25]. Random-effects models were used [26]; if continuous data were reported in median and range, estimates of mean and standard deviation were calculated using a standardized validated tool.

Evaluation of the quality of the study. The methodological quality of the studies, included in the meta-analysis, was performed using the Cochrane risk-of-bias assessment tool for RCTs [27].

## Results

### Trial identification

The search strategy identified 1919 studies, and 70 additional records were identified through other sources (as can be seen in the PRISMA diagram, Supplemental Fig. 1). After deduplication, 852 citations were screened of which 821 were excluded on the basis of title and abstract. For the remaining 31 studies, the full texts were obtained and reviewed. Sixteen studies were included [28–43], and 15 studies were excluded from the meta-analysis (three were not randomized [44–46], six RCTs performed a short follow-up [47–52], in three RCTs the timing of the follow-up was different from 6 and 12 months [53–55], two RCTs used the sutureless Trabucco technique [56, 57], and one RCT analyzed the neurectomy of the iliohypogastric nerve [58]).

The 16 RCTs included evaluated 1550 patients (Table 1) [28–43]: Among these studies, 756 patients underwent neurectomy (neurectomy group) and 794 patients underwent ilioinguinal nerve preservation surgery (nerve preservation group).

**Table 1:** Summary of studies analyzed

Author and year of publication	Nation	Time of enrollment	Number of centers	Number of inguinal hernia repair performed
Bansal 2003 [28]	India	2009–2010	1	50
Belim 2014 [29]	India	2013–2013	1	67
Crea 2010 [30]	Italy	2006–2007	1	97
Hokkam 2009 [31]	Egypt	NR	1	52
Karakayali 2010 [32]	Turkey	2004–2007	1	115
Koshmohabat 2012 [33]	Iran	2009–2010	2	140
Kudva 2015 [34]	India	2008–2009	1	90
Malekpour 2008 [35]	Iran	2005–2006	1	100
Mui 2006 [36]	China	2003–2004	1	99
Mulkipatil 2017 [37]	India	NR	1	84
Omar 2018 [38]	Egypt	2015–2016	1	40
Ravichandran 2000 [39]	United Kingdom	NR	1	40
Saravanan 2019 [40]	India	NR	1	80
Shah 2018 [41]	India	2012–2014	1	260
Shamita 2014 [42]	India	NR (one year study)	NR	56
Sharif 2019 [43]	Pakistan	2014–2015	3	180

Most of these studies were performed in Asia (12 RCTs), and only few studies were performed in Europe (2 RCTs) and Africa (2 RCTs). No study was performed in America and Australia. India published the highest number of studies (7 RCTs). Only one study was multicentric. The studies were conducted from 2003 to 2015 and published from 2006 to 2019.

### Trial quality analysis

Risk of bias in included studies. For details on the risk of bias of the included trials, please see the characteristics of included studies in Supplemental Fig. 1a and Supplemental Fig. 1b and our risk-of-bias criteria in Supplemental Table 1 (briefly, we used the classic criteria of Cochrane, with the exception in the “incomplete outcome data” risk of bias of a 15% loss of patients as threshold for high/low risk. We added two risk factors, marked below with the asterisk (*). If there was no information about the analyzed risk, we left empty spaces in the risk-of-bias table as fourth judgment for the studies).

Randomization. Random sequence generation was well explained in nine trials [28, 31, 32, 35–38, 41, 43]; therefore, these studies were considered to have a low risk of bias, with the exception of eight papers [29, 30, 33, 34, 39, 40, 42] with an unclear process of randomization.

Allocation. Of the 16 RCTs, four reported details about allocation concealment, and they were considered to have low risk of bias [29, 35, 36, 42]; one trial had an unclear reporting of allocation concealment [33]. Moreover, 11 RCTs did not report information about allocation, so they were considered as having unknown bias grade [28, 30–32, 34, 37–41, 43].

Blinding of participant and personnel. Of the 16 RCTs, seven reported details about blinding of participants and personnel. Since the studies focused on the surgical procedure, we considered that complete blinding of the personnel was impossible; therefore, we decided to only assess the blinding of the patients. These studies were considered to have low risk of bias [32–36, 39, 42]. One RCT had an unclear reporting of its blinding process [38]. Eight RCTs did not report information about blinding, so they were considered to have unknown blinding information [28–31, 37, 40, 41, 43]. However, it is not mentioned the personnel blinded in these RCTs.

Blinding of outcome assessment. Of the 16 RCTs, eight reported details about blinding of outcome assessment, and they were considered to have low risk of bias [28, 32–36, 39, 42].

Eight trials did not report information about the blinding of assessment, so they were considered to have unknown blinding information [29–31, 37, 38, 40, 41].

Incomplete outcome data. We decided to judge RCTs to have low risk of bias if outcome data were missing or missing outcome data were less than 15%, with reasons for missing outcome data unlikely to be related to the true outcome. Consequently, 13 RCTs were judged as having low risk of bias [28, 30–36, 38, 39, 41–43].

Selective reporting. One trial had no information about secondary outcomes, other than the absence of information about the protocol, marking this item empty [40]. All remaining trials had information about secondary outcomes, but had no reference to the protocol, so they were judged as having unclear risk [28–39, 41–43].

Other potential sources of bias. The Cochrane Community did not approve these last two items, so they were added for analyzing two more aspects that we judged important for the outcome of the studies.

*Comorbidities associated with worsening or ambiguity. The criteria for having “high risk” of bias were any of the following criteria, other than the local exclusion criteria: bilateral hernia, complicated hernia (incarcerated, strangulated, inflamed, recurrent).

*Procedures that may negatively affect the outcome. All studies that clearly identified the ilioinguinal nerve were judged to have “low risk” [28, 30, 32–34, 36, 38, 39, 41], and all studies in which the ilioinguinal nerve was not clearly reported were judged to have “high risk” [29, 31, 35, 37, 40, 42, 43].

### Results at 6 months after surgery

#### Postoperative groin pain

**Fig. 1:**
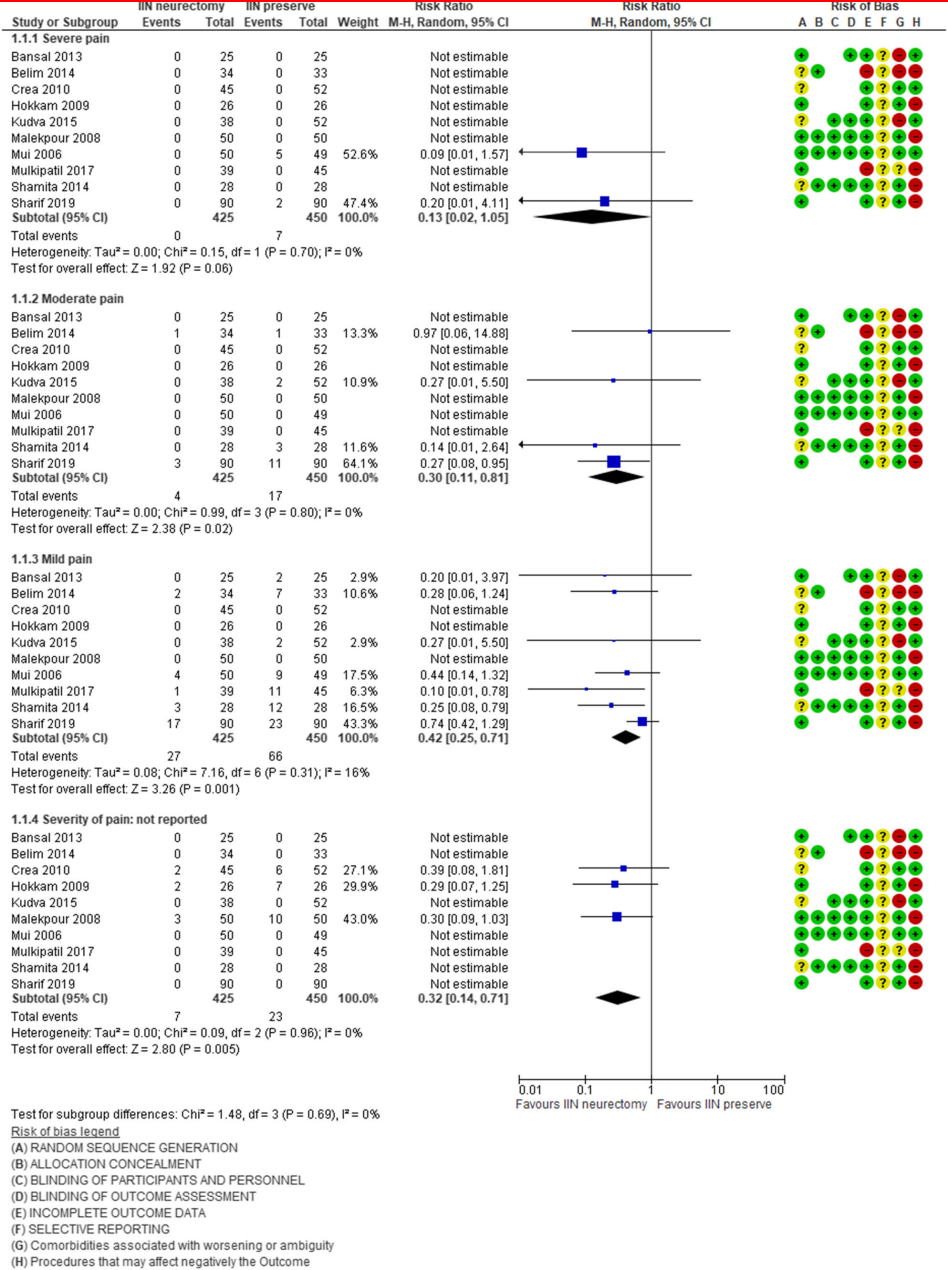
Postoperative groin pain at the 6th month after surgery

Ten studies [28–31, 34–37, 42, 43] reported postoperative pain at 6 months, including 425 patients who underwent neurectomy and 450 patients who underwent ilioinguinal nerve preservation surgery. In seven of these studies (304 neurectomy vs 322 nerve preservation), the intensity of pain was assessed in three degrees of severity: mild pain, moderate pain, and severe pain. In three studies (121 neurectomy vs. 128 nerve preservation), pain severity was not evaluated. The analysis shows a statistically significant reduction in the presence of pain 6 months after surgery at 8.94% (38/425) in the neurectomy group vs 25.11% (113/450) in the nerve preservation group [RR 0.39, 95% CI 0.28–0.54; Z = 5.60 (*P* < 0.00001)] and the heterogeneity was very low [Tau^2^ = 0.00; Chi^2^ = 7.00, df = 9 (*P* = 0.64); *I*^2^ = 0%]. The subgroup analysis shows that only mild and moderate chronic groin pains were significantly present in the neurectomy group: mild pain (RR 0.42, 95% CI 0.25–0.71), moderate pain (RR 0.30, 95% CI 0.11–0.81), and severe pain (RR 0.13, 95% CI 0.02–1.05) (Fig. 1). Only a few studies have performed a more accurate analysis of PCP; this assessment was performed based on how the groin pain manifested itself in relation to various physical activities:

1. The incidence of postoperative groin pain at rest was significantly low in the neurectomy group (RR 0.19, 95% CI 0.06–0.63) (Supplemental Fig. 3a). In this analysis, the heterogeneity between the included studies was absent [Tau^2^ = 0.00; Chi^2^ = 0.46, df = 3 (*P* = 0.93); *I*^2^ = 0%].

(2) The incidence of postoperative groin pain when performing daily activities was significantly low in the neurectomy group (RR 0.16, 95% CI 0.03–0.88) (Supplemental Fig. 3b). In this analysis, the heterogeneity between the included studies was absent [Tau^2^ = 0.00; Chi^2^ = 0.03, df = 1 (*P* = 0.87); *I*^2^ = 0%].

(3) The incidence of postoperative groin pain after moderate activities was significantly low in the neurectomy group (RR 0.18, 95% CI 0.06–0.53) (Supplemental Fig. 3c). In this analysis, the heterogeneity between the included studies was absent [Tau^2^ = 0.00; Chi^2^ = 0.28, df = 2 (*P* = 0.87); *I*^2^ = 0%].

(4) Postoperative groin pain after vigorous activities was significantly low in the neurectomy group (RR 0.22, 95% CI 0.09–0.51) (Supplemental Fig. 3d). In this analysis, the heterogeneity between the included studies was absent [Tau^2^ = 0.00; Chi^2^ = 0.22, df = 2 (*P* = 0.90); *I*^2^ = 0%].

**Fig. 2:**
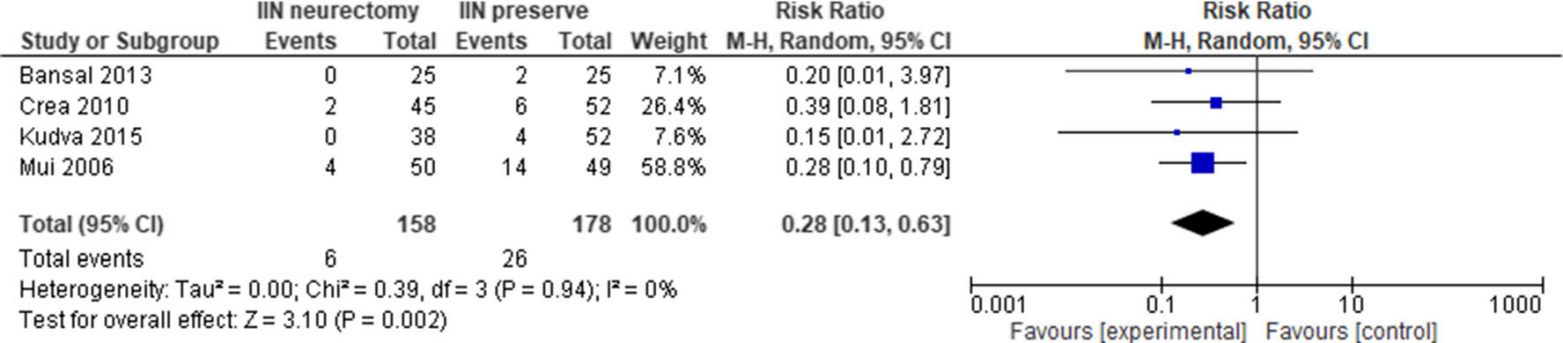
Postoperative groin pain at the 6th month in studies that identified the IIN

In order to evaluate the role of missed nerve identification during hernioplasty, we have performed another subgroup analysis of the nine studies that identified the IIN [28, 30, 32–34, 36, 38, 39, 41] only four studies [28, 30, 34, 36] reported the 6th month evaluation of pain. This new analysis shows a significant reduction of the pain in either group and the same trend in favor of neurectomy reported in the previous overall analysis: statistically significant reduction of pain 6 months at 3.79% (6/158) in the neurectomy group vs 14.6% (26/178) in the nerve preservation group [RR 0.28, 95% CI 0.13–0.63; Z = 3.10 (*P* = 0.002)]; the heterogeneity between the included studies was absent Tau^2^ = 0.00; Chi^2^ = 0.39, df = 3(*P* = 0.94); *I*^2^ = 0% (Fig. 2).

### Results at 12 months

Only two studies [30, 32] (*n* = 212) investigated the prevalence of postoperative groin pain 12 months after the intervention (100 neurectomy vs. 112 nerve preservation). These studies revealed no significant differences in the 12-month postoperative groin pain rate: 9% (9/100) in the neurectomy group vs. 17.85% (20/112) in the nerve preservation group (RR 0.50, 95% CI 0.24 to 1.05) (Supplemental Fig. 4a); the heterogeneity was absent [Tau^2^ = 0.00; Chi^2^ = 0.15, df = 1 (*P* = 0.70); *I*^2^ = 0%]. The subgroup analysis of the different severities of chronic groin pain was not carried out since no study reported data necessary for inclusion. The ilioinguinal nerve was identified in both studies; therefore, we do not report a different subgroup analysis. However, only one study carried out a more accurate analysis of postoperative chronic groin pain; this assessment was performed accounting how the groin pain manifested itself in relation to various physical activities. Regarding the occurrence of pain related to physical activities, no significant differences were noted between the two groups in terms of postoperative groin pain at rest (Supplemental Fig. 4b), during daily activities (Supplemental Fig. 4c), and after vigorous activities (Supplemental Fig. 4d).

### Paresthesia 6 and 12 months after surgery

**Fig. 3:**
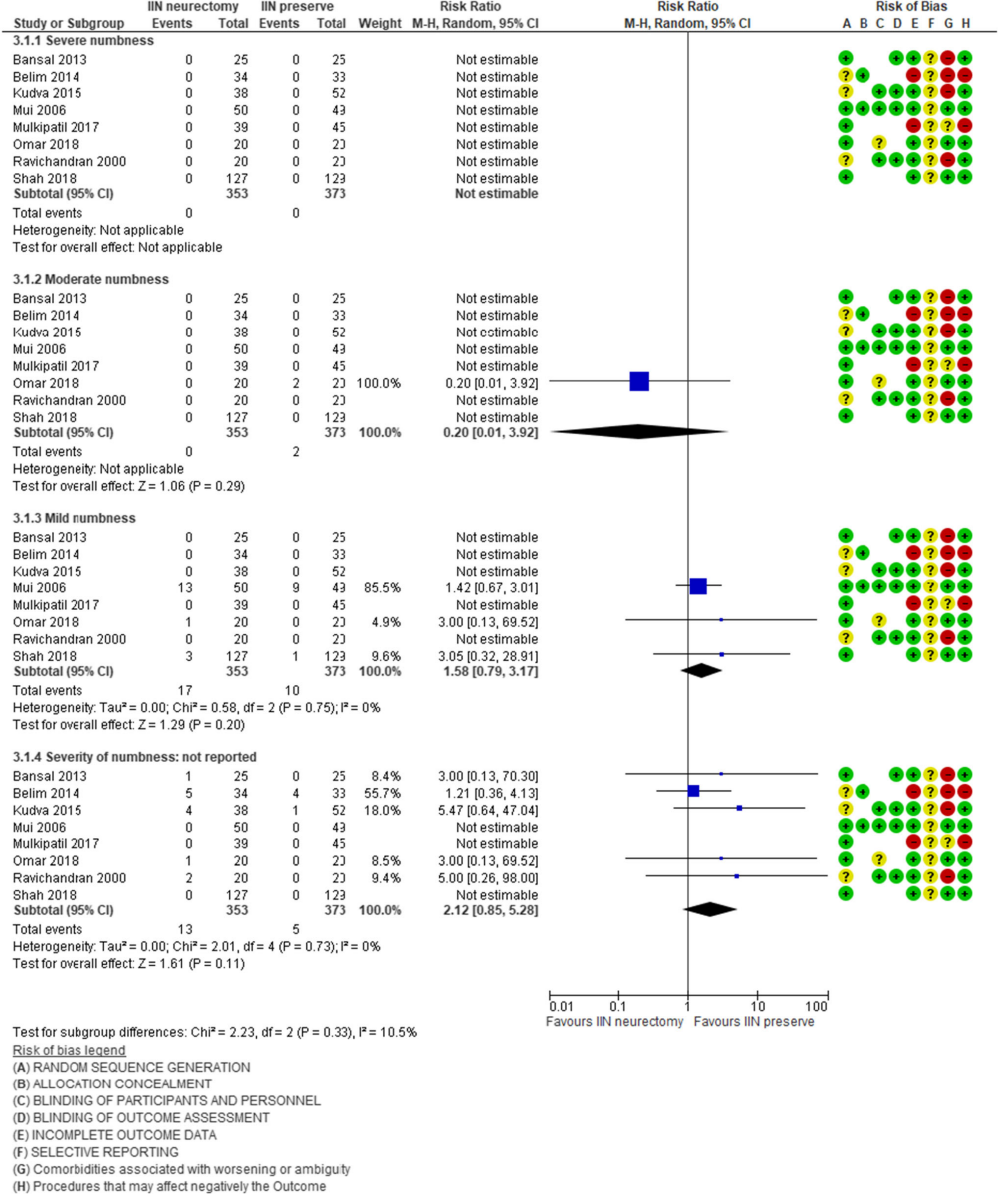
Paraesthesias 6 months after surgery

Eight studies [28, 29, 34, 36–39, 41] (*n* = 726) reported 8.5% rate of groin paresthesia at 6 months after surgery (30/353) in neurectomy group vs. 4.5% patients in the nerve preservation group (17/373). This result is positive for inguinal nerve preservation, but it is not statistically significant [RR 1.62, 95% CI 0.94–2.80] (Fig. 3); the heterogeneity was absent [aTau^2^ = 0.00; Chi^2^ = 2.90, df = 6 (*P* = 0.82); *I*^2^ = 0]. The subgroup analysis of the studies [28, 34, 36, 38, 39, 41] that identified the IIN did not report a reduction of the rate of paresthesia and reported the same trends of the previous analysis in favor of inguinal nerve preservation: RR 1.75, CI 95% 0.95–3.22, Z = 1.79 (*P* = 0.07); the heterogeneity was absent [Tau^2^ = 0.00; Chi^2^ = 2.63, df = 5 (*P* = 0.76); *I*^2^ = 0%].

Only one study [32] (*n* = 115) reported the presence of paresthesia 12 months after the intervention (7.27%, 4/55 patients in the neurectomy group vs. 5%, 3/60 patients in the nerve preservation group). The incidence of paresthesia was lower in preservation group (RR 1.45, CI 95% 0.34–6.21), but it was not statistically significant.

### Hypoesthesia 6 months after surgery

**Fig. 4:**
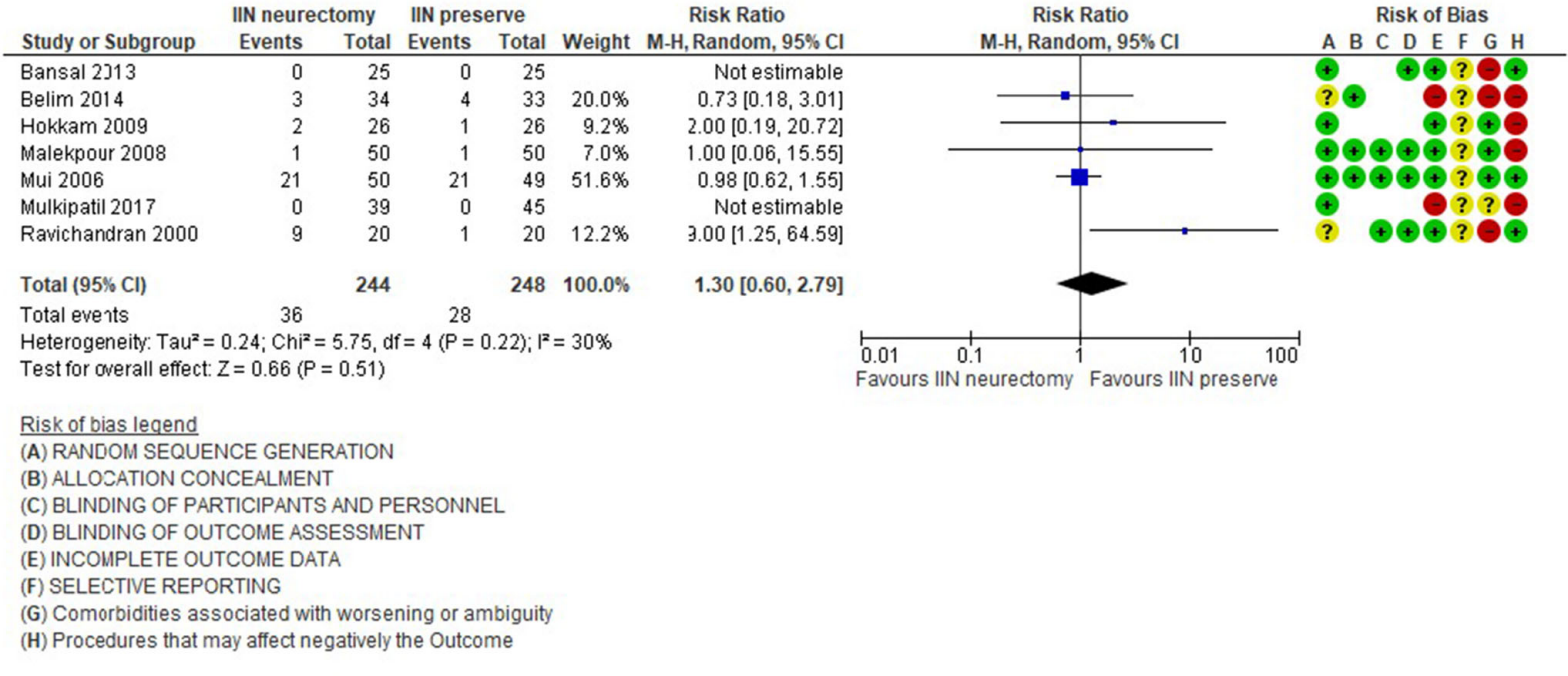
Hypoesthesia 6 months after surgery

In total, seven studies [28, 29, 31, 35–37, 39] (492 patients) reported the presence of hypoesthesia 6 months after the intervention (14.75%, 36/244 patients in the neurectomy group vs. 11.29%, 28/248 patients in the nerve preservation group). This shows that inguinal nerve preservation has a lower rate of hypoesthesia than neurectomy, but no statistically significant difference was found [RR 1.30, 95% CI 0.60–2.79, Z = 0.66 (P = 0.51)] (Fig. 4); the heterogeneity was low [Tau^2^ = 0.24; Chi^2^ = 5.75, df = 4 (P = 0.22); *I*^2^ = 30%].

The subgroup analysis of the studies [28, 36, 39] that identified the IIN reported an increase of hypoesthesia’ rate, respectively, 31.57% in neurectomy group and 23.04% in IIN preservation and reported the same trends of the previous analysis in favor of inguinal nerve preservation [RR 2.47, 95% CI 0.24, 24.96], Z = 0.77 (*P* = 0.44)]. However, the heterogeneity was very high [Tau^2^ = 2.33; Chi^2^ = 5.38, df = 1 (*P* = 0.02); *I*^2^ = 81%].

Only one study [32] (115 patients) reported the presence of hypoesthesia 12 months after the intervention (25.45%, 14/55 patients in the neurectomy group vs 11.66%, 7/60 patients in the nerve preservation group). This shows that preservation of the inguinal nerve has lower incidence of hypoesthesia, but no statistically significant difference was found.

## Discussion

This systematic review and meta-analysis allowed us to analyze a significant number of patients and included 16 RCTs that evaluated ilioinguinal nerve preservation versus neurectomy for chronic groin pain after open tension-free mesh Lichtenstein inguinal hernia repair. While this is an important topic, strength and conclusions of this analysis are completely dependent on the quality and care with which these various RCTs were done. The reality is that there is no consensus on whether to resect the ilioinguinal nerve or not during open inguinal hernia repair with mesh; this analysis is not likely to change this situation and most surgeons will continue to resect the nerve selectively. One of the limitations of this systematic review is that 75% of the studies were performed in Asia and none in the Americas or Australia. Seven of the RCTs apparently did not clearly even identify the ilioinguinal nerve, which is the principal outcome on which the study was based, so only nine papers have identified the nerve reliably in this review. As a consequence, the group of nerve preservation seemed to include the cases in which the nerve might be unknowingly incorporated into the mesh or even unconsciously sectioned by the surgeons. The incidents of hypoesthesia or paresthesia in nerve preservation group might be caused by unintentional transection of the nerve. Moreover, the chronic inguinal pain might be caused by the incorporation of the nerve into the mesh due to missed identification of the nerve during surgery. In a systematic review of literature and meta-analysis based on 5265 half-body examinations, the identification rate of the IIN was 94.4% (95% CI 89.5–97.9) [59]. The subgroup analysis of the studies in which was identified the IIN reported a lower incidence of postoperative pain at 6 months and the same trend in favor of neurectomy. This result suggests the need to perform the nerve identification to reduce the postoperative pain after open inguinal hernioplasty.

Furthermore, only ten studies reported postoperative pain at 6 months and in three of those pain severity was not evaluated. Only two studies reported postoperative pain at 12 months, with just over 100 patients in each group. In reporting pain outcomes, the high degree of heterogeneity present limits the ability to draw conclusions from the aggregated data. Prior meta-analyses have questioned the suitability of pooling data for many of these studies [8, 60, 61]. The term “post-hernioplasty chronic pain” has a wide range of interpretations in literature. The Committee of the International Association for the Study of Pain defines chronic pain as any pain reported by the patient for 3 months or longer after surgery [62]. However, with the use of a synthetic mesh for hernia repair, as a result of a reaction against the foreign material, a strong inflammatory response starts and can eventually lead to a foreign body granulomatous reaction [63] that can last up to weeks or months before developing a fibrotic/epithelioid envelope [64]. Moreover, from the analysis of the included and excluded trials in our study and the majority of papers dealing with this theme, the results were strongly positive up to 3 months, but from 6 months to 1 year after the intervention outcomes significantly varied. Therefore, in this study, we decided to include the criteria of chronic pain at 6 months (starting point) and 12 months (ending point) after a hernia surgery to analyze what would appear to be a critical period for evaluating the effectiveness of ilioinguinal nerve neurectomy.

Three prior meta-analyses of RCTs on ilioinguinal neurectomy during Lichtenstein repair have been published [8, 60, 61], including approximately half of the RCTs identified in this manuscript. These previous meta-analysis found no effect on postoperative chronic pain and did find some evidence of increased hypoesthesia. In our meta-analysis, the majority of the new studies and data comes from a relatively narrow geographic region (seven new studies from India representing the majority of patients included). This high number of RCTs performed in the same region can be associated to any local factors (e.g., types of commonly used/available mesh products) that may confound these results. Another problem of this meta-analysis is the suitability of pooling data for many of these studies.

After neurectomy, sensitivity disturbances may accompany pain or may onset separately [65] In our systematic review and meta-analysis, prophylactic neurectomy of the IIN is associated with an increased the groin paresthesia at 6 months after surgery in the neurectomy group vs nerve preservation group, but this result is not statistically significant [RR 1.62, 95%CI 0.94–2.80; Z = 0.51 (*P* = 0.61)]. Furthermore, the presence of hypoesthesia 6 months after the intervention was lower in inguinal nerve preservation group than neurectomy group, but no statistically significant difference was found (RR 1.30, 95% CI 0.60–2.79). The bias of this results is the lower number of studies included in these analyses (only half of the papers included reported data at 6 months and only one paper reported data at 12 months), as well as the heterogeneous evaluation of these data for the absence of a common standardized system. The improved use of Dermatome Mapping Test in common clinical practice can represent an adequate system for the standardization of future RCTs [66]. In the current torrent of data, cautions are needed for the lack of this additional crucial information about paresthesia and hypoesthesia.

## Conclusions

Regardless of surgical choices, the nerve identification is recommended to reduce 6^th^ month postoperative pain: in effect in both groups with nerve identification a significant reduction of the pain and a trend in favor of neurectomy group was reported.

In the context of the previously reported limitations, prophylactic ilioinguinal nerve neurectomy in Lichtenstein hernia repair seems to offer some advantages about pain in the first postoperative period. Considering paresthesia and hypoesthesia, the result was not significant, although it was mostly in favor of preservation; it might be possible that the small number of cases led to this insignificancy.

Nowadays, prudent surgeons should discuss with patients and their families the uncertain benefits and the potential risks of neurectomy before performing the hernioplasty.

Further research must be conducted, especially in the long-term period, to provide additional data that can confirm the results of this systematic review and meta-analysis. So far, we believe that in the Lichtenstein procedure, prophylactic neurectomy of the ilioinguinal nerve can be a valid choice to reduce the incidence of postoperative chronic pain.

## Supplementary Information

Supplementary file1 (DOCX 170 kb)

Supplementary file2 (DOCX 28 kb)

Supplementary file3 (DOCX 22 kb)

Supplementary file4 (DOCX 53 kb)

Supplementary file5 (DOCX 44 kb)

Supplementary file6 (DOCX 49 kb)

Supplementary file7 (DOCX 49 kb)

Supplementary file8 (DOCX 43 kb)

Supplementary file9 (DOCX 40 kb)

Supplementary file10 (DOCX 40 kb)

Supplementary file11 (DOCX 40 kb)

## Abbreviations

IIN: Ilioinguinal nerve
RR: Risk ratio
PCP: Postoperative chronic pain
RCT: Randomized controlled trial
VAS: Visual analog scale
VRS: Verbal rating scale
SDC: Supplemental digital content
CI: Confidence intervals
df: Degrees of freedom
