# Supplementary figures and images for: Ilioinguinal Nerve Neurectomy is better than Preservation in Lichtenstein Hernia Repair: A Systematic Literature Review and Meta-analysis
Source: World J Surg. 2021 Feb 19;45(6):1750–60. doi: 10.1007/s00268-021-05968-x (PMC8093155; doi:10.1007/s00268-021-05968-x)

SDC 1. Figure 1 PRISMA 2009 flow diagram


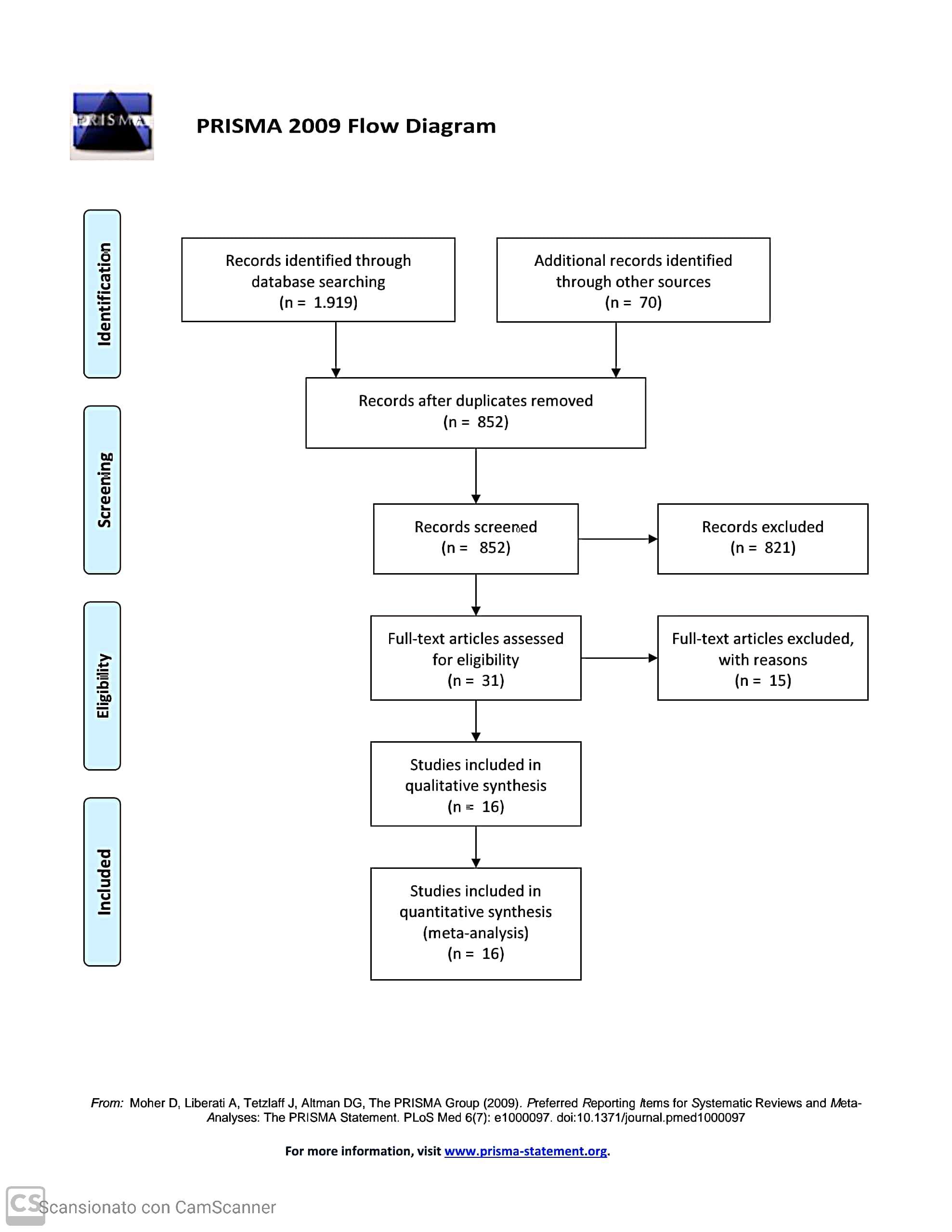


Figure 1: PRISMA 2009 flow diagram 1

Supplement: Supplementary file 1 — Supplementary file1 (DOCX 170 kb) [file 268_2021_5968_MOESM1_ESM.docx]

Supplemental Figure 3a : postoperative groin pain at rest (6th month)


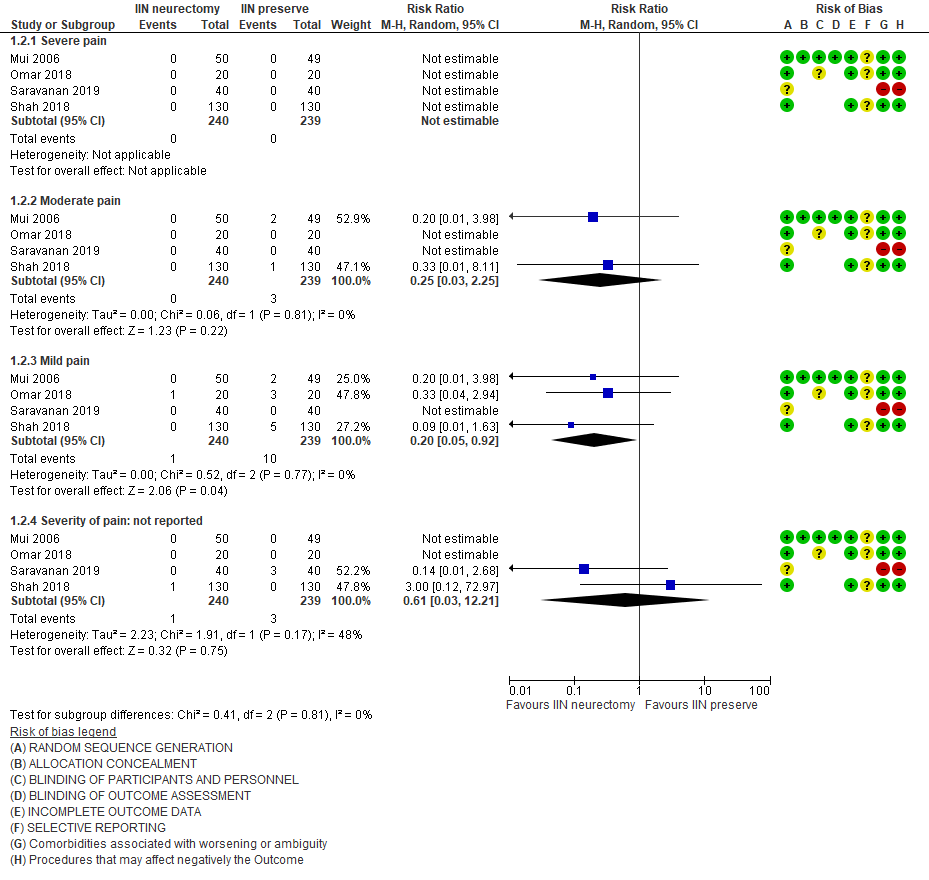

Supplement: Supplementary file 4 — Supplementary file4 (DOCX 53 kb) [file 268_2021_5968_MOESM4_ESM.docx]

Supplemental Figure 3b: postoperative groin pain when performing daily activities (6th month)


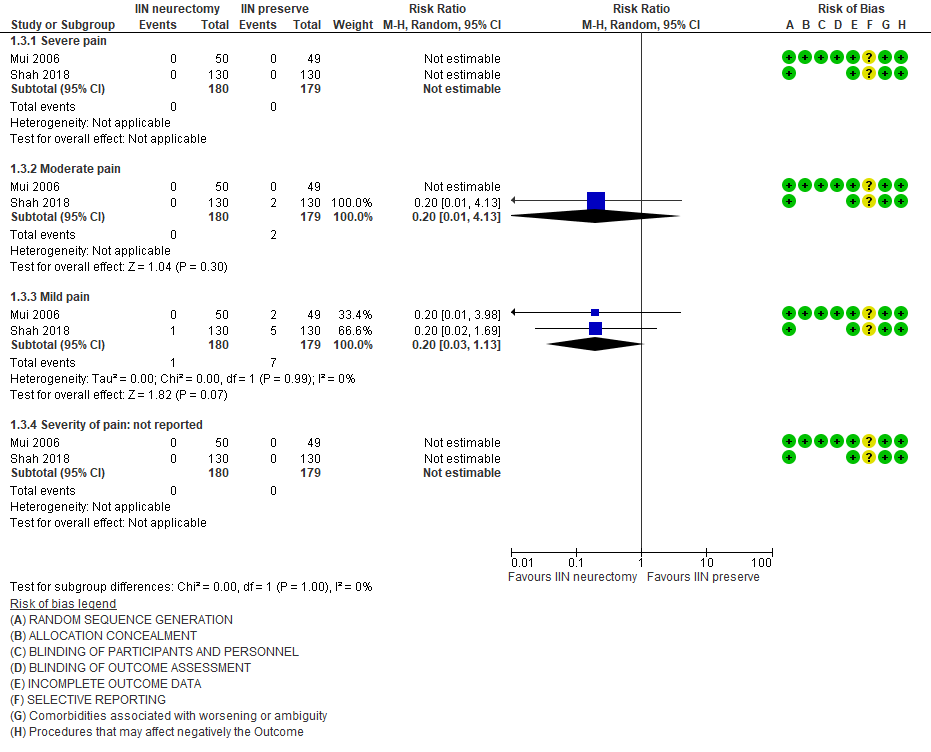

Supplement: Supplementary file 5 — Supplementary file5 (DOCX 44 kb) [file 268_2021_5968_MOESM5_ESM.docx]

Supplemental Figure 3c: postoperative groin pain after moderate activities (6th month)


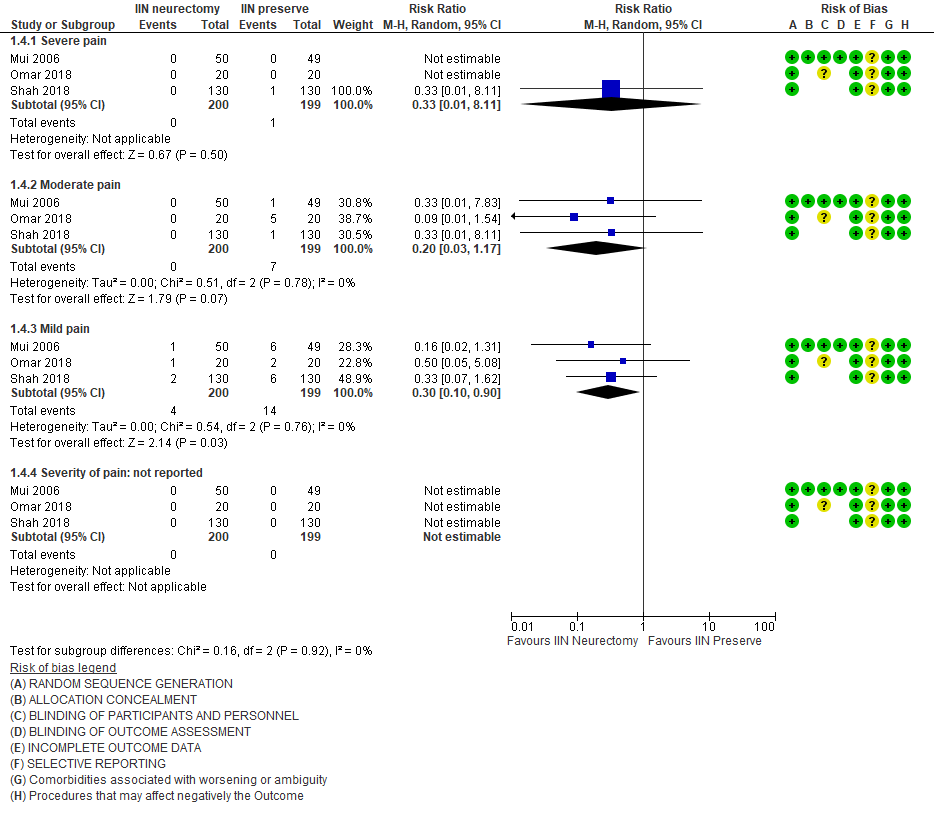

Supplement: Supplementary file 6 — Supplementary file6 (DOCX 49 kb) [file 268_2021_5968_MOESM6_ESM.docx]

Supplemental Figure 3d: postoperative groin pain after vigorous activities (6th month)


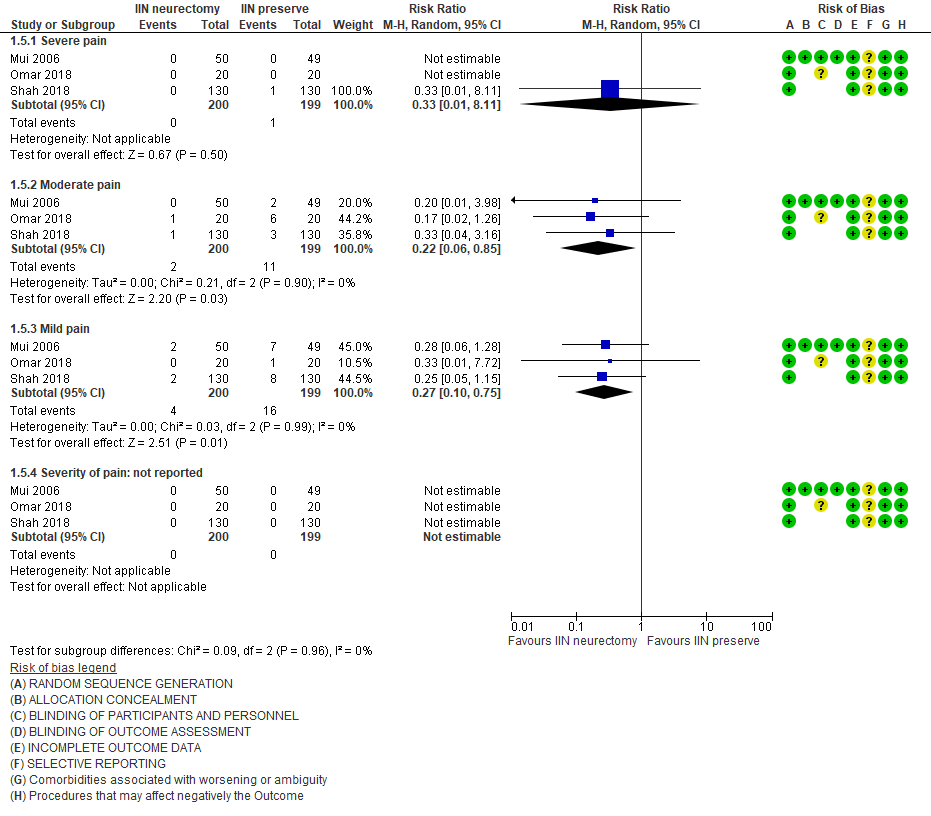

Supplement: Supplementary file 7 — Supplementary file7 (DOCX 49 kb) [file 268_2021_5968_MOESM7_ESM.docx]

Supplemental Figure 4a: Postoperative groin pain at 12th month


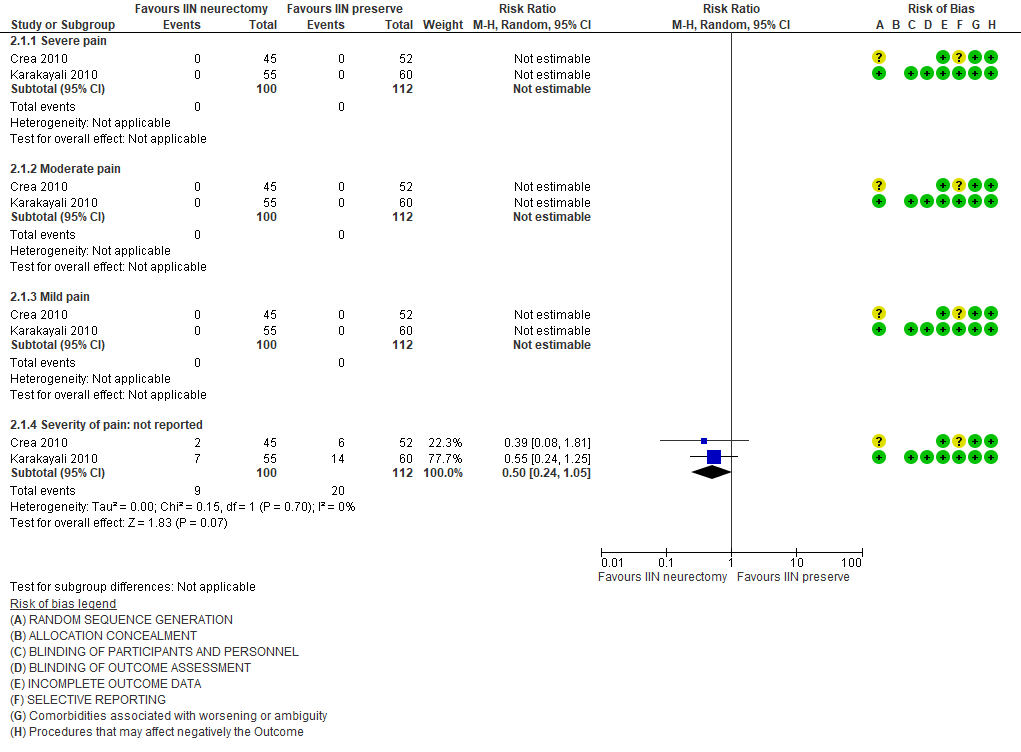

Supplement: Supplementary file 8 — Supplementary file8 (DOCX 43 kb) [file 268_2021_5968_MOESM8_ESM.docx]

### **Supplemental Figure 4b :** **“Postoperative groin pain at rest (12th month)”**


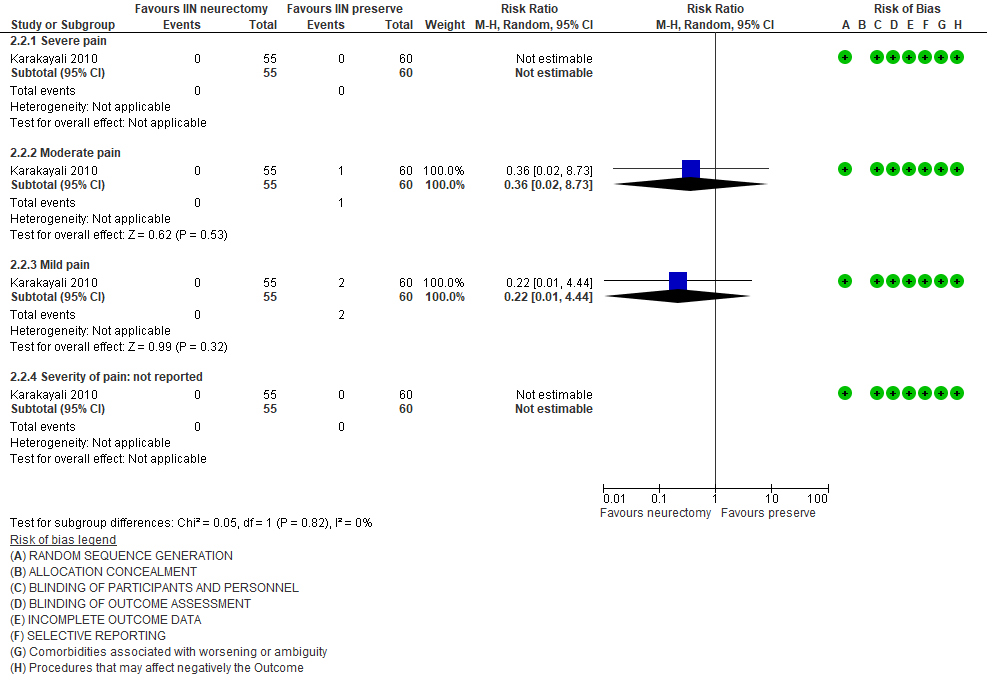

Supplement: Supplementary file 9 — Supplementary file9 (DOCX 40 kb) [file 268_2021_5968_MOESM9_ESM.docx]

### **Supplemental Figure 4c: Postoperative groin pain at daily activities (12th month)**


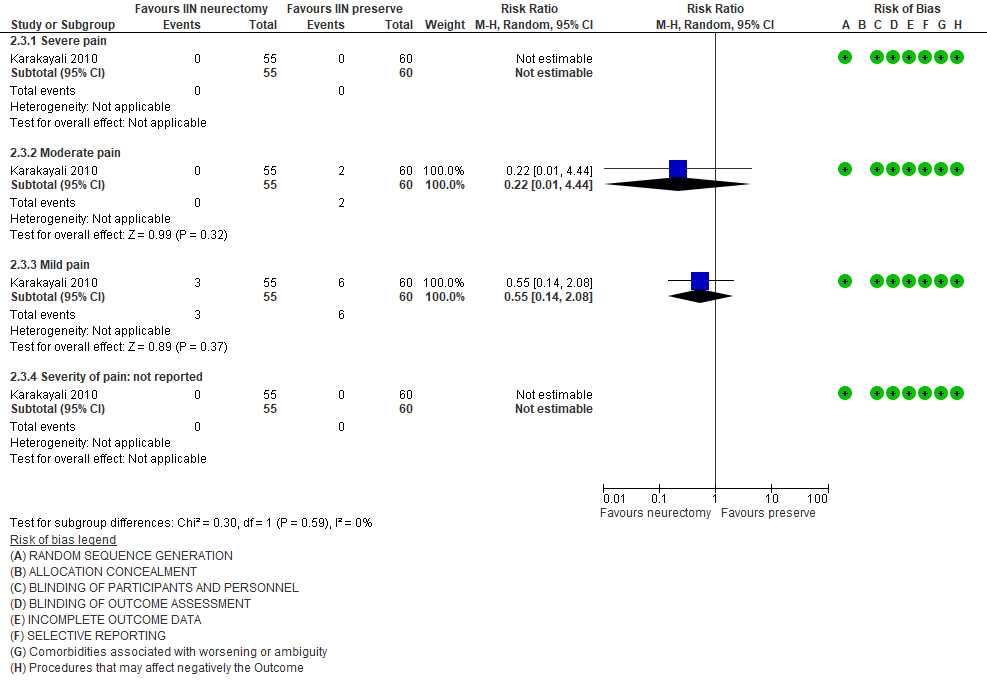

Supplement: Supplementary file 10 — Supplementary file10 (DOCX 40 kb) [file 268_2021_5968_MOESM10_ESM.docx]

### **Supplemental Figure 4d: “Postoperative groin pain after vigorous activities”**


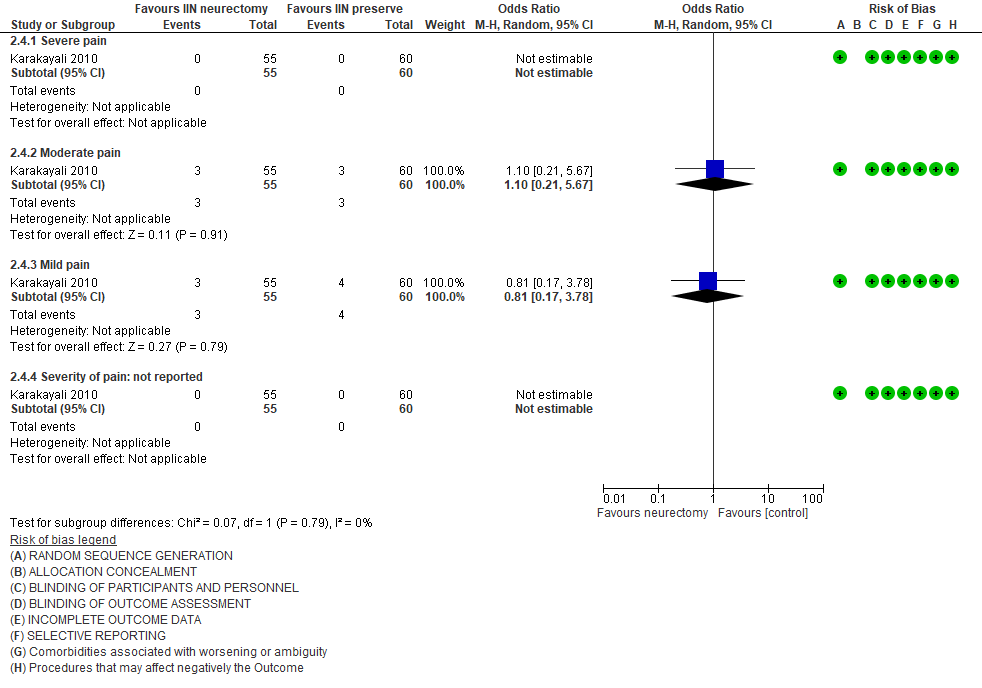

Supplement: Supplementary file 11 — Supplementary file11 (DOCX 40 kb) [file 268_2021_5968_MOESM11_ESM.docx]
