# Supplementary material for: Ilioinguinal Nerve Neurectomy is better than Preservation in Lichtenstein Hernia Repair: A Systematic Literature Review and Meta-analysis
Source: World J Surg. 2021 Feb 19;45(6):1750–60. doi: 10.1007/s00268-021-05968-x (PMC8093155; doi:10.1007/s00268-021-05968-x)

Figure 2a. Risk of bias graph: review authors' judgments about each risk of bias item presented as percentages across all included studies.


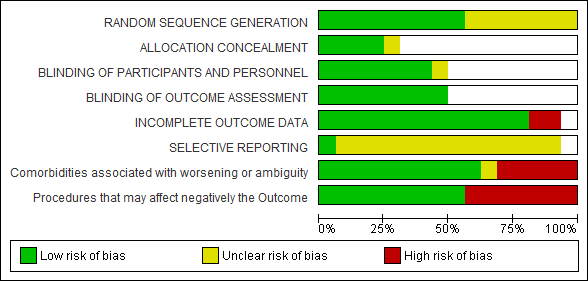


SDC 2b. Risk of bias summary: review authors’ judgments about each risk of bias item for each included study


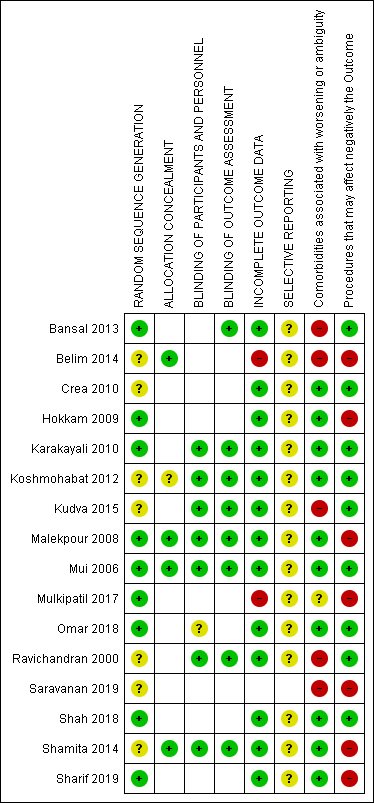

Supplement: Supplementary file 2 — Supplementary file2 (DOCX 28 kb) [file 268_2021_5968_MOESM2_ESM.docx]
